# Evaluation of a football fitness implementation initiative for an older adult population in a small-scale island society

**DOI:** 10.3389/fpubh.2024.1406601

**Published:** 2024-07-16

**Authors:** May-Britt Skoradal, Tórur Sjúrðarson, Helgi Winther Olsen, Eli Nolsøe Leifsson, Vincent Pelikan, Magni Mohr, Annika Helgadóttir Davidsen

**Affiliations:** ^1^Centre of Health Science, Faculty of Health, University of the Faroe Islands, Tórshavn, Faroe Islands; ^2^Faculty of Education, University of the Faroe Islands, Tórshavn, Faroe Islands; ^3^FSF Sport Science Unit, Faroese Football Association, Tórshavn, Faroe Islands; ^4^Faculty of Sport Science, University of Leipzig, Leipzig, Germany; ^5^Department of Sports Science and Clinical Biomechanics, SDU Sport and Health Sciences Cluster (SHSC), Faculty of Health Sciences, University of Southern Denmark, Odense, Denmark

**Keywords:** quality of life, flow, healthy aging, soccer, exercise training

## Abstract

**Background:**

The proportion of older people increases globally, which calls for sustainable interventions promoting healthy aging. Therefore, we aimed to evaluate the potential of Football Fitness as a sustainable model to promote quality of life, mental health, and physical function for older adult.

**Methods:**

The study was conducted in collaboration with a municipality, a football club, and a university, and was designed as a randomized controlled trial. A total of 66 people (34 women, 32 men) older than 60 years were randomly assigned (60/40) to a Football Fitness (FOT) (*n* = 38, 20 women, 18 men) and a control group (CON) (*n* = 28, 14 women, 14 men). FOT participated in Football Fitness for 12 weeks. Quality of life (QoL) and mental wellbeing were determined pre-and post-intervention. Physical loading and Flow experience were measured in one representative training session. Blood pressure (BP), Yo–Yo Intermittent endurance test level 1 (Yo–Yo IE1), sprint performance, postural balance, and body composition were also performed pre-and post-intervention.

**Results:**

An improvement in mental wellbeing was observed for both groups from pre- to post-intervention (*p* values <0.001) with no between-group differences. Regarding QoL, the environment domain improved in FOT compared to CON (*p* = 0.02). Mean Flow (*M* = 5.69; SD = 1.07) and perceived importance (*M* = 4.20; SD = 1.42) and average experienced difficulty was *M* = 5.23 (SD = 2.67), perceived skill (*M* = 5.23; SD = 2.56), and perceived balance (*M* = 5.64; SD = 1.56). These levels of flow can be characterized as being high. A between-group effect (*p* = 0.02) existed for systolic BP, which decreased (*p* < 0.01) by −5% [−8; −1%] in CON and remained unchanged in FOT. Both groups improved the Yo–Yo IE1 to a similar extent, with 28% [11; 44%] (*p* = 0.001) in FOT and 27% [9; 46%] in CON (*p* = 0.005). Postural balance improved (*p* = 0.004) by 38% [13; 63%] in FOT only, resulting in a superior (*p* = 0.01) balance score in FOT compared to CON post-intervention (*p* = 0.004).

**Conclusion:**

Football Fitness improved the environmental quality of life domain and postural balance in older adults. Additionally, it appears to be a feasible group activity for older adults that promotes high flow and physical loading during training.

## 1 Introduction

The world’s demographical scenery is changing with the proportion of older adults rising to a higher level than ever before. For example, it is estimated that in 2040 ~15% of the global population will have an age older than 65 years (1). Since most non-commendable diseases and chronical conditions, ranging from cardiovascular, metabolic, inflammatory, neurological, and muscle-skeletal are associated with aging, this builds several challenges in the healthcare system (2, 3). Moreover, psychiatric conditions and loneliness are also highly represented in older adults (4, 5), which calls for sustainable interventions for older adults aiming to promote healthy aging, mental health, and wellbeing.

Physical activity is a cornerstone in healthy aging (6). Group-based activities with broad-spectrum health impacts combining physical activity and social interaction, may be necessary to attract, motivate and ensure sustained participation in the older adult population. Football Fitness may qualify as a sport concept that satisfies the criteria above (7). The concept of using small-sided football games (8), as well as other team sports [see for example (9, 10)], has been shown to be highly efficient in promoting healthy aging (11) and quality of life in older adult populations (12). Moreover, several long-term trials have been conducted in populations such as hypertensive women (13), older adult women (14), older adult men with prostate cancer (15–17), as well as other older adult populations (10), with positive results that demonstrate the strength of the model in relation to sustainability.

The Faroe Islands is an archipelago consisting of 18 islands that are linked together by tunnels, bridges, or ferry connections. The population is ~55,000. The Faroe Islands have among the highest life expectancies in the world, and the altered demographical landscape is a marked obstacle to overcome for the healthcare system and especially the municipalities that are main responsible for the older adult sector. The municipalities also play a main role in financing the sports club, thus, implementing physical activity models, such as Football Fitness, appears highly feasible and relevant in a small island community, such as the Faroe Islands. We have previously, successfully implemented Football Fitness for young and middle-aged women in the Faroe Islands (18), as well as middle-aged women with a clinical condition (13), but it is unknown if similar results can be obtained with older adult men and women.

The purpose of this study is to evaluate the feasibility and effectiveness of implementing Football Fitness as a group-based physical activity model for promoting wellbeing, quality of life, and general health status among older adult individuals in the Faroe Islands. The study aims to determine whether Football Fitness can be sustainably implemented at the municipal level to address the challenges of an aging population, with a focus on its potential to enhance both physical and mental health through a combination of physical activity and social interaction. Thus, this study tests the hypothesis that Football Fitness holds a high implementation potential as a group-based physical activity model to be implemented in a sustainable manner at municipality level to promote wellbeing and quality of life, as well as general health status among older adult individuals. The implementation trial was carried out in Klaksvík, the second largest municipality in the country with a population of ~5,000 (19) corresponding to ~10% of the national population. The study is a collaboration between the municipality of Klaksvík, the local football club, the Faroese Football association, and the University of the Faroe Islands.

## 2 Methods

### 2.1 Study design

This study was initiated by one of the largest municipalities in the Faroe Islands, and was derived from the municipality’s wish to offer an appropriate exercise program for citizens older than 60 years. The research group was invited to participate in this initiative as investigators and supervisors. This study was designed as a randomized controlled trial (RCT) with one intervention group and one control group.

#### 2.1.1 Recruitment

All citizens older than 60 years living in the municipality of Klaksvík were invited to participate. The municipality staff carried out open recruitment through their social media platforms and website in the period from June 20th to August 15th 2022. The enrolled participants received written information and were invited to an information meeting. All participants provided written consent before the beginning of the study. Before the study commenced, two individuals active in the local football club received theoretical instruction and practical training in conducting Football Fitness training (20), provided by researchers from the university in collaboration with the football association.

#### 2.1.2 Inclusion criteria

All citizens in the municipality of Klaksvík over 60 years old were invited to sign up.

### 2.2 Participants and randomization

**Figure 1:**
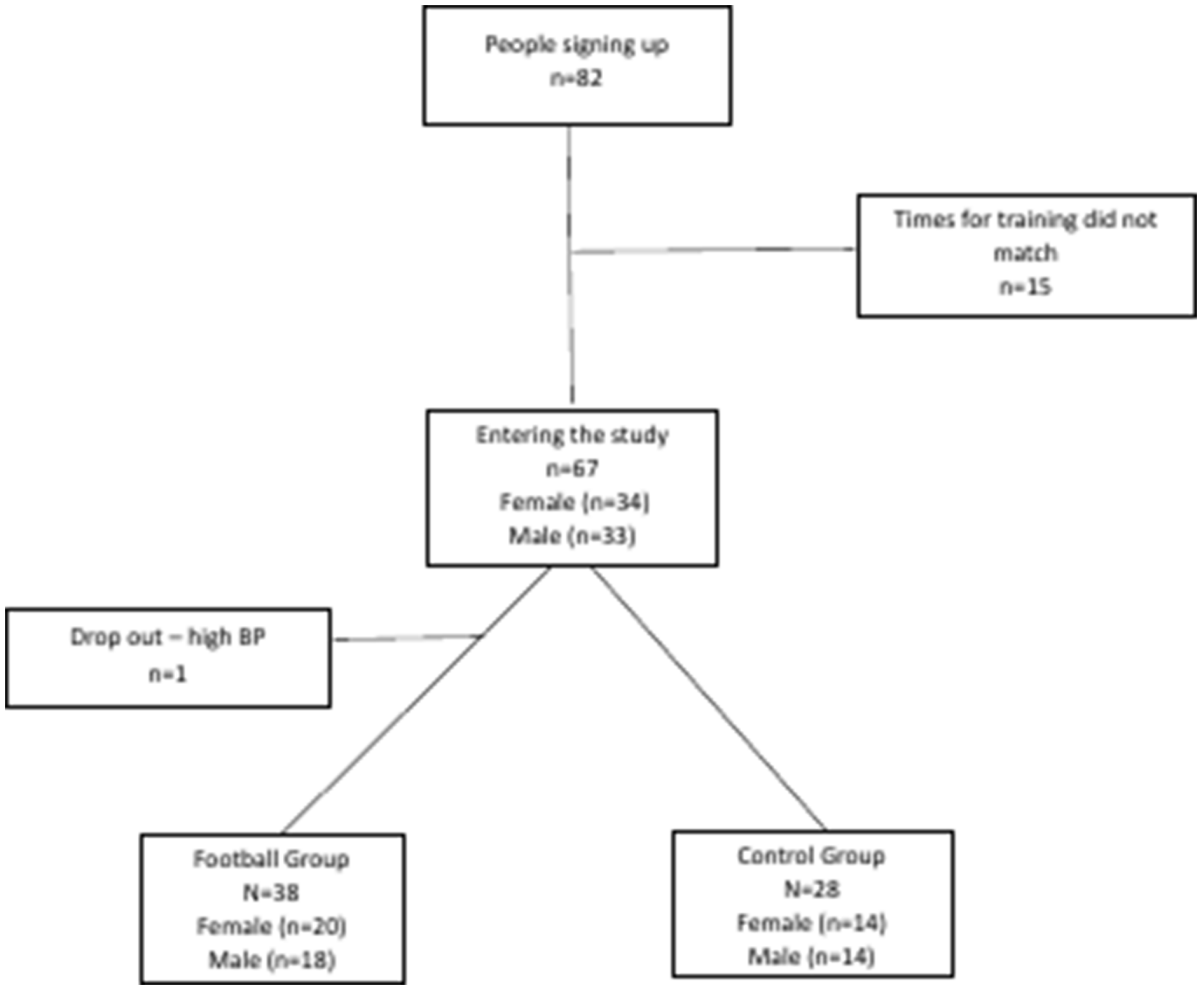
Schematic flowchart outlining recruitment, dropout, and retention of study participants for the randomized controlled trial.

A total of 82 persons signed up for the study. Fifteen participants could not participate due to the times for the training sessions, thus 67 participants entered the study. One participant dropped out during the tests; 66 participants were thus randomly assigned (60/40) to a Football Fitness group (FFG) (*n* = 38, 20 women and 18 men) or a control group (CON) (*n* = 28, 14 women and 14 men), stratified for sex and age (Figure 1).

### 2.3 Intervention

The only full-sized football field in the town was made available for the training sessions three times 1 h a week for 12 weeks, and the participants were encouraged to participate in at least two sessions per week. One training session was held early during working hours, one in the evening and one on Sunday mornings. Adherence to the planned exercise sessions averaged a total of 19 ± 8 sessions over the 12-week intervention period, corresponding to 1.6 ± 0.7 sessions per week. Each session lasted 1 h and was divided into warm-up (10–15 min), strength training (10 min), skill training and small-sided football games (35–40 min). The trainers followed a training manual that detailed all the exercises. The manual included two warm-up programs that could be used individually or combined, as well as examples of strength training exercises and technical football drills (20). The small-sided football games were organized as mixed-gender matches 4v4–7v7 on fields, the sizes of which were adjusted according to the number of participants as previously described (21, 22). After each training, the participants had the opportunity to meet in an adjacent room to socialize and drink coffee.

### 2.4 Test battery

A pre- and post-intervention battery of tests was conducted. First, as primary outcome variables, quality of life and mental wellbeing were measured. Second, physical assessments such as arterial blood pressure, resting heart rate, body weight, fat percentage, lean body mass and different markers of exercise performance were measured. Further, acute response regarding the flow and physical performance were investigated during and after a training session in week 11, respectively. All pre- and post-intervention tests were performed in the afternoon. The participants were instructed to avoid caffeine and smoking the last 60 min before the physical measurements and to avert vigorous exercise on the day of testing. First, the blood pressure, resting heart rate, and body composition were measured, and the questionnaires were completed. Hereafter, the physical tests were done in a sports hall (Table 1). All physical tests were completed on the same day.

**Table 1:** Baseline characteristics of the participants including age, body composition, Yo–Yo IE1, blood pressure, and RHR.

	Age (y)	Height (cm)	Weight (kg)	BMI (kg/m^2^)	Lean body mass (kg)	Body fat (%)	Yo–Yo IE1 test (m)	20 m-Sprint (s)	Stork balance stand test (s)	SBP (mm/Hg)	DBP (mm/Hg)	RHR (bpm)
**All participants (*n* = 66)**												
Mean	66.3	171	78.7	26.8	31.1	29.0	370	4.8	2.06	141	86	69
SD	4.5	9	14.6	4.1	7.4	8.4	212	1.0	1.32	10	10	9
**Football group (*n* = 38, female, *n* = 20, male, *n* = 18)**												
Mean	65.9	171	80.0	27.5	31.7	28.8	365	4.8	2.15	139	87	70
SD	4.7	9	14.7	4.5	7.4	8.8	231	1.2	1.48	15	9	7
**Control group (*n* = 28, female, *n* = 14, male, *n* = 14)**												
Mean	66.9	171	76.8	26.0	30.3	29.2	376	5.0	1.90	144	86	67
SD	4.32	8	14.6	3.3	7.5	8.1	187	0.8	1.11	18	11	10

#### 2.4.1 Quality of life and mental wellbeing

Quality of life was measured with a Faroese version of the abbreviated version of the WHO Quality of Life (WHOQOL-BREF). The WHOQOL-BREF includes 26 questions about different aspects of quality of life, consisting of four domains: physical health (seven items), psychological health (six items), social relationships (three items), and environmental health (eight items); and two items on general QOL and health (23, 24). Mental wellbeing was measured with a Faroese version of the Warwick-Edinburgh Mental Wellbeing Scale (WEMWBS) which is a 14-item questionnaire covering different aspects of eudaimonic and hedonic wellbeing (25, 26). Both measures have demonstrated acceptable psychometric properties (24, 27). Reliability analyses were conducted for both measures; Cronbach’s *α* for WHOQOL-BREF was 0.89 and 0.98 for WEMWBS. The trial participants filled out the questionnaires before and after the intervention.

#### 2.4.2 Acute response

During the intervention, we monitored one representative training session in week 11, to investigate the acute response regarding the physical exertion during training. To quantify the physical load, we used an integrated GPS (global positioning system) and HR (heart rate) system called Polar Teampro (Polar Electro Oy, Kempele, Finland) with GPS sampling at a 10 Hz frequency, 200 Hz tri-axial accelerometer, gyroscope, magnetometer, and HR monitor. Before the session, each participant got delivered one unit and a heart rate strap, which they had to wear throughout the session. After the session the units were synchronized and uploaded to Polar Team Pro online platform where the data was exported and analyzed by the research team. The included measurements were peak HR, average HR, time spent in 80–90% of HR max (80–90% HRmax), time spent in 90–100% of HR max (90–100% HRmax), total numbers of accelerations and decelerations (no. of acc and dec), total distance (TD), distance covered 6–8 km/h (low speed running distance: LSRD), distance covered 8–11 km/h (moderate speed running distance: MSRD), distance covered 11–13 km/h (high speed running distance: HSRD), distance covered >13 km/h (Sprint distance: SPD) these categories have been used in previous research (28). Due to the lack of information regarding the maximal heart rate of each participant, the maximal heart rate reached during the session were set a peak HR and used to calculate time spent in each HR zone.

#### 2.4.3 Flow

To assess flow [defined as a psychological state where individuals feel cognitively efficient, motivated, and happy simultaneously (29)], the Danish version of the 13-item Flow Kurz Skala (30) was utilized, which evaluates the total flow score (10 items) on a seven-point Likert scale (1 = *not at all*, 7 = *very much*). Values closer to 7 indicate very high flow rates, while values closer to 1 indicate very low flow experiences. High flow values are defined as being >5 (31). Cronbach’s alpha values of *α* = 0.90 indicate a high internal consistency of the scale. The Flow Kurz Skala also evaluates the worry component in three items, as worry can counteract the flow experience. The participants completed the questionnaires immediately at the end of a training session in week 11.

#### 2.4.4 Blood pressure and resting heart rate

Arterial blood pressure (BP) and resting heart rate (RHR) were measured in a seated position according to standard procedures (32). The BP was measured once in both arms and then repeated twice on the arm with the highest value, separated by a minute pause. The average of the three tests was recorded as the test result (32).

#### 2.4.5 Body composition

Body weight, fat percentage, and muscle mass were measured with an InBody 270 multi-frequency body composition analyzer (Biospace, CA, USA) in accordance with the manufacturer’s recommendation and other studies (33, 34).

#### 2.4.6 Testing of exercise performance

##### 2.4.6.1 Cardiorespiratory fitness

The participants completed the Yo–Yo intermittent endurance test level 1 (Yo–Yo IE1) before and after the training intervention as previously described by (35). Baseline-tests were conducted 1 week prior to the first training session and the post-test was conducted within 1 week after the last training session. The tests were conducted indoors on a polyurethane surface at environmental temperatures of 18–20°C. A standardized warm-up preceded the Yo–Yo IE1 test to minimize the risk of injuries. The pre and post-tests were conducted at the same time of the day. All participants were familiarized with the test procedure prior to the experiment as per the guidelines presented in Krustrup et al. (36).

##### 2.4.6.2 Sprint performance

After 10 min of rest, the participants completed a 20 m sprint test. The court was set with timing gates on the starting line (0 m) and at the endpoint (20 m). The participants were asked to run as fast as possible through the court. To reduce variances within the data, the gates were set at a height of 1 m at every test. The test was completed two times, interspersed by 2 min of recovery. The average 20 m sprint time was recorded as the test result.

##### 2.4.6.3 Postural balance

The postural balance test (The Standing Stork Balance Test) was used to evaluate the participants’ postural equilibrium as previously described (37). Participants stood on their preferred leg with the opposite foot resting on the knee of the standing leg while placing their hands on their waist. The test began when the participants lifted their heel from the floor to balance on the ball of the foot, and a stopwatch was controlled by the test leader. The test would end if the heel touched the floor, one or both hands moved from the waist, or the foot left the knee. Three consecutive test trials were performed on the same leg for each participant and the average value was used as the test result. Prior to the test, participants were given one practice attempt on each leg. The Standing Stork Balance Test has been demonstrated to have good reliability and is considered a valid clinical tool for measuring static balance, as it correlates (*r* = 0.65, *p* < 0.001) with other conventional balance tests like the Flamingo test (37).

### 2.5 Statistical analyses

Data are presented as means with 95% confidence intervals unless otherwise stated. The statistical analysis of all outcome measures but flow was conducted based on the intention-to-treat principle, which included all randomized participants. Statistical analysis of continuous endpoints was modeled using linear mixed method for repeated measures with fixed effects of time, group, and their interactions. The difference in response between groups was determined by the time × group interaction effect. A Sidak-adjusted post-hoc analysis was conducted for all end points to detect possible within-group time-specific differences and between-group differences at baseline and post-intervention. The mixed effect model was specified with a repeated effect of visit, a restricted maximum likelihood estimation method, the Kenward-Roger degrees of freedom method and an appropriate covariance structure. Model fit was evaluated using graphical methods. Statistical tests were two-sided, and the level of significance set to 0.05. SPSS was used for statistical analysis (IBM SPSS Statistics, version 28.0.0).

## 3 Results

### 3.1 Quality of life and mental wellbeing

Results showed no effect between the groups regarding mental wellbeing. There was, however, a significant improvement in mental wellbeing for both groups from pre to post (*p* values <0.001). There was no effect between the groups regarding quality of life (*p* values >0.05), besides the environment domain, where the experimental group improved significantly compared to the control group (*p* = 0.02) (Table 2).

**Table 2:** Values are presented as means with 95% confidence intervals from a repeated measures linear mixed model with time, group, and time × group as fixed factors.

Outcome	Intervention				Time × group
	CON		FOT		
	Pre	Post	Pre	Post	
WEMWBS	54.0 [51.7; 56.3]	57.4 [55.1; 59.7]**	52.7 [50.8; 54.7]	56.9 [55.0; 58.9]**	*p* = 0.523
Physical health	16.2 [15.3; 17.0]	16.3 [15.4; 17.1]	16.9 [16.2; 17.6]	16.5 [15.8; 17.2]	*p* = 0.40
Psychological health	16.5 [15.7; 17.2]	16.9 [16.2; 17.7]	16.3 [15.7; 16.9]	16.6 [16.0; 17.2]	*p* = 0.75
Social relationships	16.4 [15.7; 17.1]	16.7 [16.0; 17.4]	15.6 [15.0; 16.2]	16.0 [15.4; 16.6]	*p* = 0.71
Environmental health	16.8 [16.4; 17.3]	16.7 [16.2; 17.1]	16.6 [16.2; 16.9]	17.0 [16.6; 17.4]	*p* = 0.02

WEMBS, Warwick Edinburgh Mental Wellbeing Scale. **Indicates a significant change from pre to post (*p* < 0.01).

### 3.2 Acute responses

Acute response measurements showed a large variation in physical exertion during a training session. Average heart rate of 131 bpm (range: 99–157 bpm) with 17 ± 09.12 and 15 ± 12.55 min spent in 80–90% and 90–100% of peak heart rate. Participants covered a total distance of 3,131 m (range: 2,402–4,014 m), including 581 ± 142 m (LSRD), 159 ± 94 m (HSRD) and 158 ± 131 (SPD). Furthermore, the participants performed on average 538 ± 86 decelerations and 520 ± 75 accelerations (Table 3).

**Table 3:** Values are presented as average with range for acute response during a training session.

	Peak HR (bpm)	Avg HR (bpm)	80–90% peak HR (tt:mm:ss)	90–100% peak HR (tt:mm:ss)	Total distance (m)	LSR (m)	MSR (m)	HSR (m)	Deceleration (no.)	Acceleration (no.)	*n*
Average	162 [123:192]	131 [99:157]	00:17:49 [00:00:00: 00:35:04]	00:15:23 [00:00:00: 00:40:12]	3,131 [2,402:4,014]	922 [256:1,482]	142 [26:321]	79 [3:250]	538 [373:655]	520 [354:625]	21

### 3.3 Flow

Of the initial 26 participants, 4 (15%) did not fully complete the questionnaire and were excluded from the data analysis. The mean flow (*M* = 5.69; SD = 1.07) and perceived importance (*M* = 4.20; SD = 1.42) reported in this study can be characterized as high. The average experienced difficulty was *M* = 5.23 (SD = 2.67), perceived skill (*M* = 5.23; SD = 2.56), and perceived balance (*M* = 5.64; SD = 1.56).

Multiple linear regression analysis was conducted to test if experienced difficulty, perceived skill, and the interaction of both variables predicted flow. The variables were centered before calculating the interaction term. The overall regression was statistically significant (*R*^2^ = 0.36), *F*(3, 18) = 3.30, *p* = 0.04. There was a significant main effect for perceived skill (*β* = 0.60, *t*(21) = 2.78 *p* = 0.01) but not for experienced difficulty (*β* = 0.04, *t*(21) = 0.20 *p* = 0.85). The interaction of experienced difficulty and perceived skill was not significant (*β* = −0.22, *t*(21) = −1.15, *p* = 0.26).

An additional regression analysis was conducted on flow with perceived balance and squared perceived balance as predictors. Perceived balance was centered before being squared. The overall regression was not significant (*R*^2^ = 0.08), *F*(2, 19) = 0.78, *p* = 0.47. Descriptive statistics of the direct measure of perceived balance show tendencies for flow being higher when perceived balance is low, just right, or too high (Table 4).

**Table 4:** Mean flow values for each value of perceived balance.

Direct measure of balance								
Too low				Just right				Too high
1	2	3	4	5	6	7	8	9
–	–	6.4 (1)	5.75 (4)	5.89 (7)	5.65 (4)	4.85 (4)	–	6.37 (3)

A potential moderation effect of the perceived importance on the relationship between the perceived balance and flow was analyzed. The overall model was not significant (*R*^2^ = 0.30), *F*(5, 16) = 1.40, *p* = 0.28.

### 3.4 Clinical investigations

#### 3.4.1 Blood pressure and resting heart rate

A time × group effect (*p* = 0.02) existed for SBP, which decreased by −5% [−8; −1%] in CON and remained unchanged in FOT (Table 5). No between-group or within-group effects were observed for DBP or RHR (Table 5).

**Table 5:** Values are presented as means with 95% confidence intervals from a repeated measures linear mixed model with time, group, and time × group as fixed factors.

Outcome	Intervention				Time × group
	CON		FOT		
	Pre	Post	Pre	Post	
SBP (mmHg)	145 [139; 152]	138 [132; 145]**	139 [134; 145]	141 [135; 147]	*p* = 0.02
DBP (mmHg)	87 [83; 90]	84 [81; 88]	86 [83; 90]	87 [84; 90]	*p* = 0.11
RHR (bpm)	67 [64; 71]	67 [64; 71]	69 [66; 72]	69 [66; 72]	*p* = 0.89
**Body composition**					
BMI	25.9 [24.3; 27.5]	25.8 [24.2; 27.4]	27.5 [26.1; 28.9]	27.3 [26.0; 28.7]	*p* = 0.91
LBM (kg)	28.9 [26.1; 31.7]	29.5 [26.8; 32.3]	31.4 [28.9; 33.8]	31.4 [28.9; 33.8]	*p* = 0.16
Total fat (%)	29.1 [25.9; 32.3]	28.8 [25.6; 32.0]	29.5 [26.7; 32.3]	29.5 [26.7; 32.3]	*p* = 0.65
**Physical performance**					
Yo–Yo IE1 (m)	384 [262; 505]	489 [368; 611]**	389 [283; 495]	497 [391; 602]**	*p* = 0.97
20 m sprint (s)	5.1 [4.6; 5.5]	4.9 [4.5; 5.3]	4.8 [4.4; 5.2]	4.8 [4.4; 5.1]	*p* = 0.69
Balance (s)	2.0 [1.4; 2.7]	2.1 [1.5; 2.8]	2.3 [1.7; 2.8]	3.2 [2.6; 3.7]**,^#^	*p* = 0.10

The result of the post-hoc analysis is indicated by **p < 0.01 for within-group change from baseline and ^#^*p* < 0.05 compared with CON. SBP, systolic blood pressure; DBP, diastolic blood pressure; RHR, resting heart rate; BMI, body mass index; LBM, lean body mass; Yo–Yo IE1, Yo–Yo intermittent endurance test level 1.

#### 3.4.2 Body composition

No statistical within-group or between-group effects were observed for any of the obtained measures of body composition (Table 5).

#### 3.4.3 Physical performance

The Yo–Yo IE1 performance improved to a similar extent in both groups, with magnitudes of 28% [11; 44%] (*p* = 0.001) in FOT and 27% [9; 46%] in CON (*p* = 0.005) (Table 5), whereas 20 m sprint time remained unchanged in both groups. Finally, postural balance improved (*p* = 0.004) by 38% [13; 63%] in FFG only, resulting in a statistically (*p* = 0.01) superior postural balance in FOT compared to CON post-intervention (Table 5).

## 4 Discussion

The purpose of this study was to test the effect of a 12-week Football Fitness intervention on the mental wellbeing, quality of life, flow, physical outcomes and exercise capacity of men and women aged 60 years and older. Other studies have found beneficial effects of Football Fitness on multiple health outcomes, including psychosocial and physical for different age groups (9, 38, 39). The main finding of this study is that Football Fitness is a feasible and easily implemented group-based physical activity model for older adult, and that this type of training appears to provide a high physiological stimulus and promotes the experience of flow measured midway in the intervention. Moreover, the intervention improved of the participants’ quality of life within the environmental domain and augmented postural balance.

Unexpectedly, the intervention did not improve the mental wellbeing of participants in the FOT group, nor did it improve three of four QOL-dimensions. There was, however, a significant change in mental wellbeing for both groups from pre to post. These findings can be partly understood through the limitations of this study, such as recruitment of participants. Since the trial was a community-based intervention, we used a potentially biased recruitment strategy by inviting all citizen in the municipality to participate. Participation thus required actively reaching out to the organizers, and it is therefore plausible that the study participants were socially and psychologically better-functioning than the general population and thus entered the trial with a higher level of functioning, introducing a ceiling-effect (40). This explanation is supported by a mean WEMWBS pre score of 54 for the CON group and 52.7 for the FOT, which is above the population mean score in Denmark, Iceland, and England (27). The result showing a significant change in mental wellbeing in both groups can be explained by a tendency of participants in CON to take up physical training. Although they were instructed to maintain their usual daily activities, some of the participants in the CON group took up physical exercise during the trial period, because their motivation for entering the trial was to be part of the FOT group. Though this is a positive effect of the trial on the individual health status, it could have diminished group differences. We do not, however, have data supporting this explanation.

The environmental domain of the WHOQOL-BREF comprises, e.g., questions about physical safety and security, the opportunity for leisure activities, and opportunities for acquiring new information and skills (23). Maintaining a decent quality of life involves being challenged both mentally and socially, being able to perform everyday household tasks, and feeling safe in one’s home surroundings. This is especially important for older adults, who may be in risk of accidents such as falling or fear of falling at home, particularly if they live alone (41). Studies have indicated that fear of falling can have a detrimental impact on quality of life, and for older adults, falls can be a serious health concern. Falls are mainly caused by environmental factors, such as bathing or ascending stairs (41–43). Postural balance has also been associated with risk of falling among older adults (44, 45). The fact that the environmental domain of the WHOQOL-BREF and postural balance significantly improved in this study suggests that Football Fitness can enhance important aspects of older adults’ physical safety and security in their home surroundings.

In this study the phenomenon of flow (31) was investigated among the individuals in the FG immediately after one training session at the end of the intervention period. The mean flow and perceived importance reported can be characterized as high. The results in this study resemble what was seen in the investigation of inactive adults who played recreational football for 16 weeks (29, 46). It involves complete absorption in an activity, disregarding time, fatigue, and other distractions (47). This rewarding experience motivates individuals to seek the activity again, influencing future engagement. Therefore, the flow score in this study may suggest that the participants will choose football playing as a leisure activity after the study period. In support of this, Football Fitness is now established in Klaksvik, and a substantial number of the participants in the present study are still taking part in the football training. This is in line with several of our other trials with middle-aged and older adult, that started out as short-term RCT’s, but developed into long-term intervention, because the participants wanted to keep on playing [see (13, 15)]. Collectively, football organized as small-sided games appears to be a sustainable physical activity model in older adult. A qualitative study reports that the biggest barriers to being physically active are that it’s expensive and access to facilities is poor (48). Contrary, Football Fitness continued to be offered to older adult in the municipality of Klaksvík. The activity was clearly advertised, with registration being relatively inexpensive and easy to access, thus making it an easily accessible form of exercise.

Postural balance improved by 38% in FFG which was significantly higher than in CON. At cross-sectional level, postural balance has been shown to be better in older individuals who have played football lifelong compared to sedentary older men and young individuals (49). Also, among young, healthy, untrained men, effects on postural balance have been observed after 12 weeks of football training (50) and for women treated for breast cancer (51). Thus, our results confirm previous research, and indicated that football training only ~1.5 times weekly for 12 weeks also upregulates balance in older adult.

It is well known that physiological aging causes a decrease of 5–10% in maximal oxygen uptake per decade (52), and an inactive lifestyle exacerbates this condition. Studies have shown a clinically relevant improvement in cardiorespiratory fitness after 12–16 weeks of Football Fitness (38, 53). Indeed, at meta-analysis level a 3.5 mL/min/kg corresponding to 1 MET increase in maximal oxygen uptake has been shown after 12–16 weeks of football training (38). In this study, both groups showed an improvement in Yo–Yo IE1 performance of 28 and 27%, respectively. Several other randomized controlled studies have shown greater improvements in football groups compared to non-exercising control groups (54), also for older adult populations (17, 22). The reason for improvement in both groups in this study is unclear, but may partly be due to a learning effect, since no familiarization was done to the Yo–Yo IE1 test and/or that some of the controls may have taken up other exercise types during the intervention period. The latter explanation is indicated by a very large improvement in some of the individuals in the control group.

The results from our study did not show significant improvement in arterial blood pressure, Yo–Yo IE1 performance, body composition, or 20 m sprint test in the football group compared to the control group. On the other hand, there was a decrease in systolic blood pressure in the control group. These findings are surprising and contradict most previous findings and meta-analysis data showing a beneficial effect on most of the physical outcome measures (53, 54). These unexpected findings can be partly associated to the limitations of this study, such as implementation, modification of the intervention, and potentially sample size (40, 55). Firstly, the average training attendance was only ~1.5 sessions weekly, which is less than the by manual recommended training volume of 2–3 weekly sessions (53, 56). Secondly, the training intensity for some participants may have been lower than expected, which also is supported by the GPS data (data not shown). According to the original protocol, teams in training sessions were intended to be mixed gender. This arrangement proved ineffective, especially because some women expressed fear of the relatively forceful shots and speed exhibited by the men. Consequently, the teams were modified so that men, and women who desired it, trained together, while the remaining women formed a separate group. Research has indeed indicated that mix-gender small-sided recreational team handball is more demanding for middle-aged/older adult women compared to their male counterparts. Men also exhibited higher cardiovascular, and locomotion demands when playing in same-gender matches, a trend opposite to that observed in women (57). The group divisions may therefore have reduced the training’s effectiveness, as it did not yield effects like those observed in comparable studies (22, 53, 58, 59). Finally, medication status was self-reported at the commencement of the study, introducing information bias. Participants did not report changes made to their blood pressure medication by their general practitioner in cases where blood pressure levels were elevated. It is conceivable that the intervention group anticipated a reduction in blood pressure during the intervention period, leading them to refrain from seeking medical consultation, in contrast to the control group.

In our trial, we have not conducted calculations separately for men and women, nor have we conducted correlation analyses between the degree of attendance, quality of life, physiological capacity, and flow. This could be an obvious avenue for future studies of the same kind. Future studies should further adopt a qualitative approach to explore participants’ experiences with this type of physical activity. Previous research indicates that football enhances social capital among inactive women (60) and fosters social interaction among men (61). The presence of social elements in training highlights the interconnectedness of health, emphasizing its importance for patients, doctors, policymakers, and researchers (60, 61).

One aim of this study was to evaluate the implementation potential of Football Fitness in a municipality in a small country for an older adult population. An endeavor that we have been successful in, previously, for middle-aged women (13, 18). After the study period, the football club, and the municipality, have been offering Football Fitness for the older adult in the north region of the Faroes twice yearly and currently more than 30 older adult have been recruited. Moreover, the applied Football Fitness model has been used as a “best practice” approach to initiate similar set-ups elsewhere in the country. In the Faroe Islands, as in other Scandinavian countries, the main responsibility for the older adult sector is at municipality level. Thus, using a community-based approach including different stakeholders (municipalities, sports clubs, national sports associations, etc.) in cooperation with the research community (university, health sector, etc.) appears as a highly efficient method to implement physical activity models for older adult populations that can be sustained.

### 4.1 Limitations

This study has several limitations that must be acknowledged. Firstly, the recruitment strategy may have introduced selection bias, as the enrolled participants might have been more physically, socially, and psychologically better-functioning than the general population, potentially leading to a ceiling effect in the outcomes obtained. Secondly, the average training attendance was only half of the recommended three sessions per week (approximately 1.5 sessions per week), which could have affected the efficacy of the training intervention in improving physiological health markers and quality of life.

Additionally, the initial mixed-gender training sessions had to be modified due to some women’s discomfort with the forceful play by men. Consequently, some women formed a separate group, potentially reducing the training’s effectiveness compared to other studies that maintained mixed-gender sessions. Another limitation is the self-reported medication status at the study’s start, and participants were not asked to report any changes to their blood pressure medication during the study period, which may have introduced information bias. Indeed, it is possible that participants in the intervention group expected their blood pressure to improve due to the intervention and thus did not seek medical consultation, unlike the control group.

Furthermore, the study did not conduct gender-specific subgroup analyses due to a lack of statistical power. These analyses could provide more detailed insights into possible gender effects and should be considered for future studies. Finally, the relatively low sample size, in conjunction with the Sidak-adjusted correction for multiple testing, might have led to an increased risk of type II errors, possibly resulting in the failure to detect actual effects in some outcomes.

## 5 Conclusion

In conclusion, the study demonstrates that football training for 12 weeks improves the environmental quality of life domain as well as postural balance in an older adult population. Moreover, the Football Fitness model is a feasible physical activity for older adults, which induces a high flow experience and physical loading.

## Data availability statement

The raw data supporting the conclusions of this article will be made available by the authors, without undue reservation.

## Ethics statement

The studies involving humans were approved by Scientific Ethics Committee, Faroe Islands. The studies were conducted in accordance with the local legislation and institutional requirements. The participants provided their written informed consent to participate in this study.

## Author contributions

M-BS: Conceptualization, Data curation, Funding acquisition, Investigation, Methodology, Project administration, Resources, Supervision, Validation, Visualization, Writing – original draft, Writing – review & editing. TS: Data curation, Formal analysis, Investigation, Methodology, Software, Writing – original draft, Writing – review & editing. HO: Investigation, Validation, Writing – original draft, Writing – review & editing. EL: Formal analysis, Investigation, Methodology, Software, Writing – original draft, Writing – review & editing. VP: Formal analysis, Methodology, Writing – original draft, Writing – review & editing. MM: Conceptualization, Funding acquisition, Investigation, Methodology, Resources, Writing – original draft, Writing – review & editing. AD: Conceptualization, Data curation, Formal analysis, Investigation, Methodology, Project administration, Software, Supervision, Writing – original draft, Writing – review & editing.
